# Engagement of Two Distinct Binding Domains on CCL17 Is Required for Signaling through CCR4 and Establishment of Localized Inflammatory Conditions in the Lung

**DOI:** 10.1371/journal.pone.0081465

**Published:** 2013-12-05

**Authors:** Sandra Santulli-Marotto, Ken Boakye, Eilyn Lacy, Sheng-Jiun Wu, Jennifer Luongo, Karl Kavalkovich, Ana Coelho, Cory M. Hogaboam, Mary Ryan

**Affiliations:** 1 Janssen Research & Development, Spring House, Pennsylvania, United States of America; 2 Department of Pathology, University of Michigan, Ann Arbor, Michigan, United States of America; University Heart Center Freiburg, Germany

## Abstract

CCL17 (TARC) function can be completely abolished by mAbs that block either one of two distinct sites required for CCR4 signaling. This chemokine is elevated in sera of asthma patients and is responsible for establishing inflammatory sites through CCR4-mediated recruitment of immune cells. CCL17 shares the GPCR CCR4, with CCL22 (MDC) but these two chemokines differentially affect the immune response. To better understand chemokine mediated effects through CCR4, we have generated chimeric anti-mouse CCL17 surrogate antibodies that inhibit function of this ligand *in vitro* and *in vivo*. The affinities of the surrogate antibodies for CCL17 range from 685 pM for B225 to 4.9 nM for B202. One antibody, B202, also exhibits weak binding to CCL22 (K_D_∼2 µM) and no binding to CCL22 is detectable with the second antibody, B225. *In vitro*, both antibodies inhibit CCL17-mediated calcium mobilization, β-arrestin recruitment and chemotaxis; B202 can also partially inhibit CCL22-mediated β-arrestin recruitment. Both B202 and B225 antibodies neutralize CCL17 *in vivo* as demonstrated by reduction of methacholine-induced airway hyperreactivity in the *A. fumigatus* model of asthma. That both antibodies block CCL17 function but only B202 shows any inhibition of CCL22 function suggests that they bind CCL17 at different sites. Competition binding studies confirm that these two antibodies recognize unique epitopes that are non-overlapping despite the small size of CCL17. Taking into consideration the data from both the functional and binding studies, we propose that effective engagement of CCR4 by CCL17 involves two distinct binding domains and interaction with both is required for signaling.

## Introduction

The homeostatic chemokine, CCL17 (TARC) has been associated with human diseases affecting various organs such as ulcerative colitis (UC), atopic dermatitis (AD), idiopathic pulmonary fibrosis (IPF) and asthma [1]–[6]. In mice, CCL17 has been linked with various inflammatory conditions presumably by setting the stage for a Th2 response through recruitment of CCR4+ immune cells, from controlling schistosomiasis and colitis to conditions of chronic pulmonary inflammation seen in fibrosis and asthma models. [7]–[13]. Neutralization of CCL17 by treatment with antibody ameliorates the impacts of disease in both the *A. fumigatus* and ova models of asthma, and liver damage in the *P. acnes* mouse model of induced hepatic injury by blocking influx of T cells. [8], [10], [11].

CCL17 functions through CCR4 which is shared with only one other ligand, CCL22 (MDC), and CCR4 interaction with each chemokine produces distinct outcomes. [14], [15]. A contributing factor may be in the differences in binding affinity; CCL22 binds CCR4 more tightly and induces receptor internalization more readily than CCL17 [14], [16]–[18]. Their pattern of expression also differs in that CCL22 production is limited to immune cells whereas CCL17 production has been reported to be expressed by many different cell types including non-immune cells [3], [19]–[22]. Differences are apparent in mediating immune function as well. For example, in the murine cecal ligation and puncture (CLP) model of experimental sepsis CCL22 promotes innate immunity whereas CCL17 seems to interfere and in some circumstances contribute to organ damage [23]. In the mouse model of pulmonary invasive aspergillosis CCL22 plays a protective role in the innate anti-fungal response whereas CCL17 plays the role of suppressor [12]. These two chemokines can play contrasting roles in establishing localized inflammation due to differential effects on Treg homeostasis in that Treg recruitment is favored by CCL22 but not CCL17 [9], [24], [25]. A role for CCL17 in contact hypersensitivity (CHS) has been established using CCL17–EGFP mice in which CCL17 expression is disrupted by insertion of the EGF coding region [21]. In these mice, CCL17 is a major factor in initiating the inflammatory response driving contact hypersensitivity (CHS) to challenge with either FITC or DNFB. A complete knock out of CCL17 function in these mice also permitted overall enhanced survival of cardiac allografts compared to heterozygous mice having one functional CCL17 allele. An alternate approach has been to use CCR4 knockout (KO) mice; however, this mutation inhibits both CCL17 and CCL22 function making it impossible to delineate the relative contribution of each chemokine [26], [27]. Aside from KO mice, the use of CCR4 antagonists in mouse models has yielded some insight; however, this does not provide a means for studying the function of the individual chemokines and overall targeting of CCR4 may introduce a new set of variables since it is also expressed on platelets [28], [29]. To further understanding of how each of these chemokines contributes to the immune response requires the ability to target them individually with the exclusive specificity afforded by neutralizing antibodies.

In order to specifically focus on the role of CCL17 in allergic airway disease we generated monoclonal surrogate antibodies and expressed them as chimeric molecules having rat V_L_ and V_H_ fused with mouse IgG1 Fc. Studies blocking CCL17 *in vivo* are reported in the literature and these studies have been conducted using commercially available polyclonal antibodies or monoclonal rat anti-CCL17 antibody, as in the murine *A. fumigatus* model of invasive lung disease [12]. To study the effects of inhibiting CCL17 function *in vivo*, we have generated rat:mouse (IgG1) chimeric mAbs and demonstrated that upon binding CCL17 they inhibit signaling mediated through CCR4 interaction [30]. These antibodies were generated using the antigen-binding region of hybridomas from rats immunized with CCL17 and fused to mouse IgG1 Fc. Chimeric mAbs were characterized for binding specificity and ability to inhibit CCL17 function as previously described [30]. Two antibodies were chosen for further study based on their unique characteristics compared to the rest of the antibodies in this panel. One of the antibodies, B202, is the only antibody we isolated that also binds mouse CCL22 even though animals were not immunized with this antigen. B202 also exhibits cross-species reactivity in that it inhibits both mouse and human CCL17 function. In contrast, B225 was chosen because it is specific for mouse CCL17 and consistently performed well *in vitro*. Treatment with either antibody resulted in reduced airway hyperresponsiveness (AHR) in the *A. fumigatus* mouse model of allergic asthma which indicates CCL17 function is neutralized *in vivo*. Although B202 binds both CCL22 and CCL17 the interaction with CCL22 is very weak having a binding affinity in the µM range. Biochemical characterization of the CCL17/CCL22 dual reactive and CCL17-specific binding antibodies reveals that the two antibodies are recognizing non-overlapping epitopes, yet both are effective at inhibiting CCL17 function. These results suggest at least two distinct binding domains are involved in CCL17 mediated signaling through CCR4 and blocking either of the binding domains is sufficient to ablate CCR4 signaling. We propose that CCL17 and CCL22 interact with a common site on CCR4 through a binding domain conserved on both chemokines. A second domain, unique to CCL17, must be concurrently engaged in order for CCL17 to induce a functional signal through CCR4.

## Materials and Methods

### Ethics Statement

Prior approval for animal work at the University of Michigan was obtained from the University Committee on Use and Care of Animals at the University of Michigan. Animals at Janssen R&D were maintained in a facility approved by the American Association for Accreditation of Laboratory Animal Care in accordance with current regulations and standards of the U.S. Department of Agriculture. The protocol was reviewed and approved by the Centocor Institutional Animal Care and Use Committee.

### Reagents

HEPES STERILE 1 M, AMRESCO (Solon, OH); MSD Blocker A MSD, (Gaithersburg, MD); MSD Read Buffer T (4×), MSD SULFO-TAG, 150 nM, rat anti-mouse CCL17 monoclonal antibody MAB529, R&D Systems (Minneapolis, MN).

### Expression of muCCL17 in E. coli BL21 (DE3)

CCL17 protein was expressed in *E. coli* then isolated from inclusion bodies and refolded to generate functional chemokine as previously described [30]. Briefly, inclusion bodies were collected in solubilization buffer consisting of 8 M urea, 5 mM EDTA, 20 mM Tris HCl, pH 7, 10 mM DTT. Solubilized inclusion bodies were clarified by centrifugation at 4°C at 18,000×g for 10 minutes then loaded onto an SPFF Colum. Protein was eluted using a gradient of 0–100% Buffer A (10 mM potassium phosphate, pH 6.8, 8 M urea) plus 1 M NaCl. Pooled fractions were refolded by dilution into refolding buffer for a final concentration of 0.1 M NaHCO_3_, 1.5 M guanidine HCl, 3 mM cysteine and 0.3 mM cystine then incubation at room temperature for 48 hours with gentle stirring followed by incubation in cold room for 66 hours.

### Hybridoma Generation

Hybridomas were generated by fusion of splenocytes prepared from rats (CD1) immunized with purified recombinant mouse CCL17 as described previously [30]. The rats were maintained in a facility approved by the American Association for Accreditation of Laboratory Animal Care in accordance with current regulations and standards of the U.S. Department of Agriculture.

Hybridoma culture supernatants were by screened for CCL17 binding by capture ELISA [30]. Briefly, 96 well ELISA plates ThermoFisher Scientific (Waltham, MA) were coated with goat anti-Rat IgG (Fc) Fab’2 Jackson ImmunoResearch (West Grove, PA) and incubated overnight at 4°C. Wells were blocked using 0.4% BSA/PBS then washed 3×with 0.2% Tween 20/PBS before addition of hybridoma supernatant followed by incubation for 30 minutes. Following wash with 0.2% Tween 20/PBS either biotinylated CCL17 or CCL22 was added and allowed to incubate for 30 minutes at room temperature. Streptavidin-HRP Jackson ImmunoResearch (West Grove, PA) was used as the detection reagent as in the presence of OPD substrate Sigma-Aldrich (St. Louis, MO). The reaction was stopped by addition of 4 N H_2_SO_4_ then plates were read at 490 nm.

### Calcium Flux Assays

The calcium mobilization assay using the T-lymphoblastic leukemia cell line, CCRF-CEM, (ATCC #: CCL-119) was performed as previously described [30]. Briefly, cells were cultured in RPMI with GlutaMAX; 10% FBS; 10 mM Hepes, 1 mM Sodium Pyruvate, 4500 mg/L glucose, and 1500 mg/mL Sodium bicarbonate at 37°C incubator with 5% CO_2_. The cells were harvested by centrifugation then resuspended at 1.6×10^6^ cells/mL then seeded in 384-well poly-D-lysine coated black view assay plate Greiner Bio-One (Monroe, NC) in the presence of Fluo-8 NW dye-loading buffer ABD Bioquest, Inc. (Sunnyvale, CA) which consists of: 10 ul Fluo-8 NW, 1 mL 10×Pluronic F127 Plus and 19 mL assay buffer with 0.1% BSA which was allowed to incubate at 37°C incubator with 5% CO_2_ for 30 min. Purified hybridoma α-CCL17 mAbs, MAB529 control mAb R&D Sytems, (Minneapolis, MN), rat IgG2_A_ isotype control MAB006 R&D Systems, (Minneapolis, MN) or assay buffer was pre-incubated with mCCL17 for 60 min at room temperature. Calcium mobilization was assessed using FDSS 6000 Hamamatsu (Bridgewater, NJ) to measure the fluorescence intensity using at 490 nm excitation and 525 nm emission.

### Determination of Binding Affinities

Biacore experiments were performed using a Biacore 3000 optical biosensor GE Healthcare Biacore AB, (Uppsala, Sweden). A Biacore biosensor surface was prepared by coupling goat anti-mouse IgG Fcγ fragment specific Ab to the carboxymethylated dextran surface of a CM-5 chip using the manufacturer instructions for amine-coupling chemistry. The coupling buffer was 10 mM sodium acetate, pH 4.5. About 5000 response units (RU) of Ab were immobilized in each of four flow cells. Kinetic experiments were run in BRB (PBS pH 7.4, supplemented with 3 mM EDTA, 0.005% Tween 20 and 400 µg/mL BSA) at 25°C. The anti-CCL22 mAbs were captured (150–230 RU) onto the anti-mouse Fcγ antibody-modified sensor chip surface. Capture of anti-CCL22 mAbs was followed by injection of murine CCL22 in solution (3.9–4000 nM in 4-fold dilutions). The association was monitored for 4 minutes in all experiments. The dissociation was monitored for 10 minutes. Regeneration of the sensor surface was obtained using a pulse of 100 mM H_3_PO_4_.

Data were processed using the Scrubber software, version 1.1 g (BioLogic Software). Double reference subtraction of the data was performed by subtracting the curves generated by buffer injection from the reference-subtracted curves for analyte injections to correct for buffer contribution to the signal and instrument noise [31]. After data processing, the data were analyzed using a simple 1∶1 binding model for steady-state affinity analysis or for off-rate determination.

For measurements obtained via Solution Equilibrium Affinity (SEA) analysis, 6-fold serial dilutions of either CCL17 or CCL22 starting 2 µM were prepared in Tris-Based Saline buffer containing 0.05% Tween-20, TBST (Thermo Scientific). Equal volumes of either mAb B202 or mAb B225 at 200 pM were added to each dilution in duplicate then incubated at 4°C for 48 hours. MSD-Streptavidin High Bind MA6000 96-well plates Meso Scale Discovery (Gaithersburg, MD) were blocked with TBST for 2 hours at room temperature then washed 3 times with MSD Wash buffer Meso Scale Discovery (Gaithersburg, MD). Biotinylated mouse CCL17 was then added to the MSD-SA plates at 25 ng/mL and incubated at room temperature for 1 hour then washed three times before addition of either the mCCL17/mAb mixtures or mCCL22/mAb mixtures then incubated for 1 hour. Plates were then washed three time and ruthenylated F(ab’)_2_ donkey anti-mouse IgG (H+L) was added and incubated for 30 minutes with moderate shaking at room temperature. After washing three times, MSD Read Buffer T with Surfactant Meso Scale Discovery (Gaithersburg, MD) prepared according to manufacturer’s directions was dispensed to each well and analyzed with a SECTOR Imager 6000 Meso Scale Discovery (Gaithersburg, MD).

### ELISA Binding of Mouse CCL17 with mAbs

MSD high bind plates Meso Scale Discovery (Gaithersburg, MD) were coated directly with 10 µg/mL recombinant mouse CCL17 proteins in 0.1 M HEPES buffer (pH 7.4) then blocked with 5% MSD Blocker A buffer for 2 hours at room temperature. After three washes with 0.1 M HEPES buffer pH 7.4, MSD 2-fold serial dilutions of Sulfo-tag labeled anti-mouse CCL17 antibodies were added (from 100 nM to 50 pM) then incubated for 2 hours with shaking at room temperature. Plates were then washed 3 times with 0.1 M HEPES buffer (pH 7.4) before addition of MSD Read Buffer T followed by analysis using a SECTOR Imager 6000 Meso Scale Discovery, (Gaithersburg, MD).

Additionally, recombinant mouse CCL17 proteins were assayed by capture ELISA where mAb, B225 (10 µg/mL) was added to each well and incubated for 2 hours at room temperature. The plate was then blocked with 5% Blocker A, and subsequently washed with of 0.1 M HEPES Buffer. Recombinant mouse CCL17 protein (20 µg/mL in 0.1 M HEPES) was added and allowed to incubate for 1 hour at room temperature. After washing 3 times, 2-fold serial dilutions of labeled anti-mouse CCL17 antibodies (from 2.5 nM to 20 pM) were added to the plate and incubated for 2 hour with shaking at room temperature. The plate was washed and read using MSD 4T read buffer as described above.

### Competition ELISA

Competition binding assays were performed to evaluate differential binding epitopes of α-mouse CCL17 antibodies. Recombinant mouse CCL17 (10 µg/mL) was directly coated on MSD HighBind plates for 2 hours at room temperature then blocked with 5% MSD Blocker A buffer for an additional 2 hours at room temperature. Plates were washed 3×with 0.1 M HEPES buffer, pH 7.4, followed by the addition of the mixture of 10 nM labeled B225 or MAB529 antibody along with appropriate competitor antibody. Labeled antibody was incubated with increasing concentrations of competitor, from 1 nM to 2 µM, and then added to the designated wells. After incubation with gentle shaking at room temperature 2 hours, plates were washed 3×with 0.1 M HEPES buffer (pH 7.4). The plate was washed and read using MSD 4T read buffer as described above.

### Antibody Cloning and Expression

The V_H_ and V_L_ region genes were cloned from each of the rat hybridomas expressing α-mouse CCL17 antibodies as described previously [30]. The chimeric mAbs were expressed as mouse IgG1/κ antibodies in HEK 293 F cells; purification of the chimeric mAbs was performed using MabSelectSuRe GE Healthcare Bio-Sciences AB, (Uppsala, Sweden) and AKTAxpress followed by elution using 0.1 M sodium acetate pH 3.5 and subsequent dialysis into DPBS pH 7.2. Endotoxin was removed using ActiClean resin Sterogene Bioseparations Inc., (Carlsbad CA). Quality and purity was assessed by SDS-PAGE and SEC HPLC TSKgel BioAssist G3SWXL, TOSOH Bioscience (King of Prussia, PA).

### Chemotaxis

The neutralizing properties of the B202, B225, CNTO5516 (control rat:mouse chimeric IgG1/κ), and MAB529 (R&D Systems) were assessed in a chemotaxis assay with EL4 cells. Chemotaxis was induced using mCCL17 and the migration of EL4 cells through a 5-µm filter was assessed using a 96-well chemotaxis plate (Neuro Probe, Inc.) Ligand and/or antibody were added to the lower chamber and the antibody concentrations ranged from 0.25 µg/mL up to 10 µg/mL. EL4 cells were washed with PBS and suspended in RPMI containing 0.1% BSA at a density of 0.5×10^6^ cells/mL, and 70 µl of this cell suspension was added to the upper chambers. The chemotaxis plates were incubated for 60 min in a 5% CO_2_-humidified incubator at 37°C, after which cells migrating across the membrane into the lower chamber were measured using the Cell Titer-Glo Luminescence Cell Viability Assay.

### β-arrestin Assay

The β-arrestin recruitment assay was performed as described previously using human CCR4 expressing CHO cells according to the manufacturer recommendations (PathHunter eXpress β-arrestin Human and Ortholog GPCR assay, DiscoveRx) [30]. Mouse CCL17 was pre-incubated in the presence or absence of: anti-CCL17 (B225), dual reactive antibody (B202), control anti-CCL17 (R&D systems) or anti-CCL22 (R&D systems) for 20–30 minutes at 37° with 5% CO_2_ before addition to cells. Following addition to the cells, the assay was allowed to proceed for 90 minutes. Chemiluminescent detection substrates were added and incubated at room temperature for 60 minutes. Luminescence readings were taken on the Perkin Elmer, Victor^3^ V Wallac1420; multilabel counter and analyzed on GraphPad Prizm software. For dose curves, concentration started at 1 µM with a 1∶2 dilution series. For antibody inhibition studies, mouse CCL17 and mouse CCL22 were used at a constant concentration of 30 nM and 20 nM, respectively.

### Chronic Fungal Asthma in Mice

Female, C57BL/6 mice (6 to 8 weeks of age) were purchased from Taconic Farms (Germantown, NY) and sensitized with *Aspergillus fumigatus* (*A. fumigatus*) antigens as previously described in detail [32]. Prior approval for mouse use was obtained from the University Committee on Use and Care of Animals at the University of Michigan. After Aspergillus sensitization, mice were challenged via i.t. instillation with live, germinated *A. fumigatus* conidia. Immediately prior to and every second day thereafter, groups of mice received one of the following by i.p. injection: 1) control IgG; 2) B225 (at 200 µg/kg or 2 mg/kg); or 3) B202 (at 200 µg/kg or 1 mg/kg) after conidia challenge. At day 7 after i.t. administration of *A. fumigatus* conidia, AHR was assessed in all groups of mice using a Buxco™ plethysmograph (Buxco, Troy, N.Y., USA). Briefly, sodium pentobarbital (Butler, Columbus, Ohio, USA; 0.04 mg/g of mouse body weight) was used to anesthetize mice prior to their intubation and ventilation with a Harvard pump ventilator (Harvard Apparatus, Reno, Nev., USA). Once baseline airway resistance was established, 210 µg/kg and 420 µg/kg of methacholine were administered i.v. through a tail vein and AHR was monitored for approximately 2 min. The peak increase in airway resistance was then recorded. After the assessment of AHR, whole lung lobes were dissected from each mouse and snap frozen in liquid nitrogen for genomic and proteomic analysis or fixed in 10% formalin for histological analyses after either hematoxylin/eosin (H/E) or Periodic acid-Schiff (PAS) staining.

### Statistical Analysis

All results are expressed as mean ± standard error of the mean (SEM). Unless otherwise stated, groups were comprised of 5 mice. A one-way ANOVA and a Student-Newman-Keuls Multiple Comparison Post Test were used to reveal statistical differences between the control group and the anti-CCL17 mAb treatment groups at day 7 after the *A. fumigatus* conidia challenge; P<0.05 was considered statistically significant.

## Results

### Surrogate Anti-CCL17 Antibodies Expressed as Rat-mouse Chimeric Antibodies Inhibit CCL17 Function in vitro

**Figure 1:**
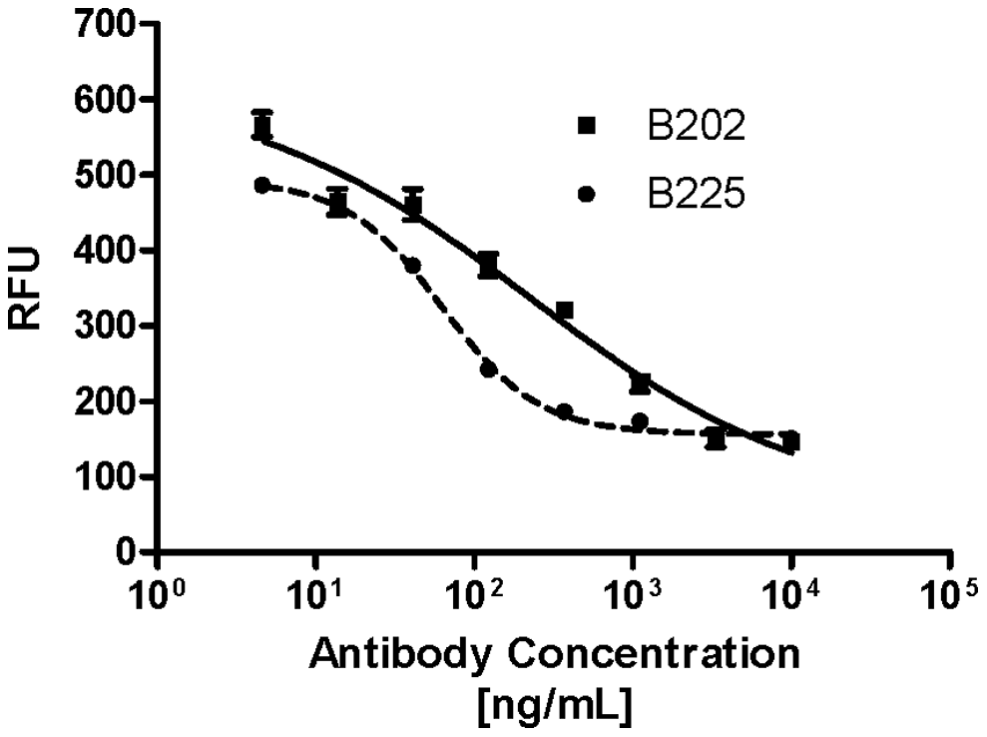
Surrogate anti-CCL17 antibodies neutralize mouse CCL17 calcium mobilization in a dose-dependent manner. CCRF-CEM cells were treated in quadruplicate with mouse CCL17 at 2 ng/mL in the presence of either B202 (*solid line/squares*) or B225 (*dotted line/circles*) with each antibody at a starting concentration of 10 µg/mL. The IC_50_ of each antibody was calculated using GraphPad Prism non-linear regression analysis and determined to be as follows: B225 63.05 ng/mL; B202 225.4 ng/mL. Results are representative of 3 experiments.

**Figure 2:**
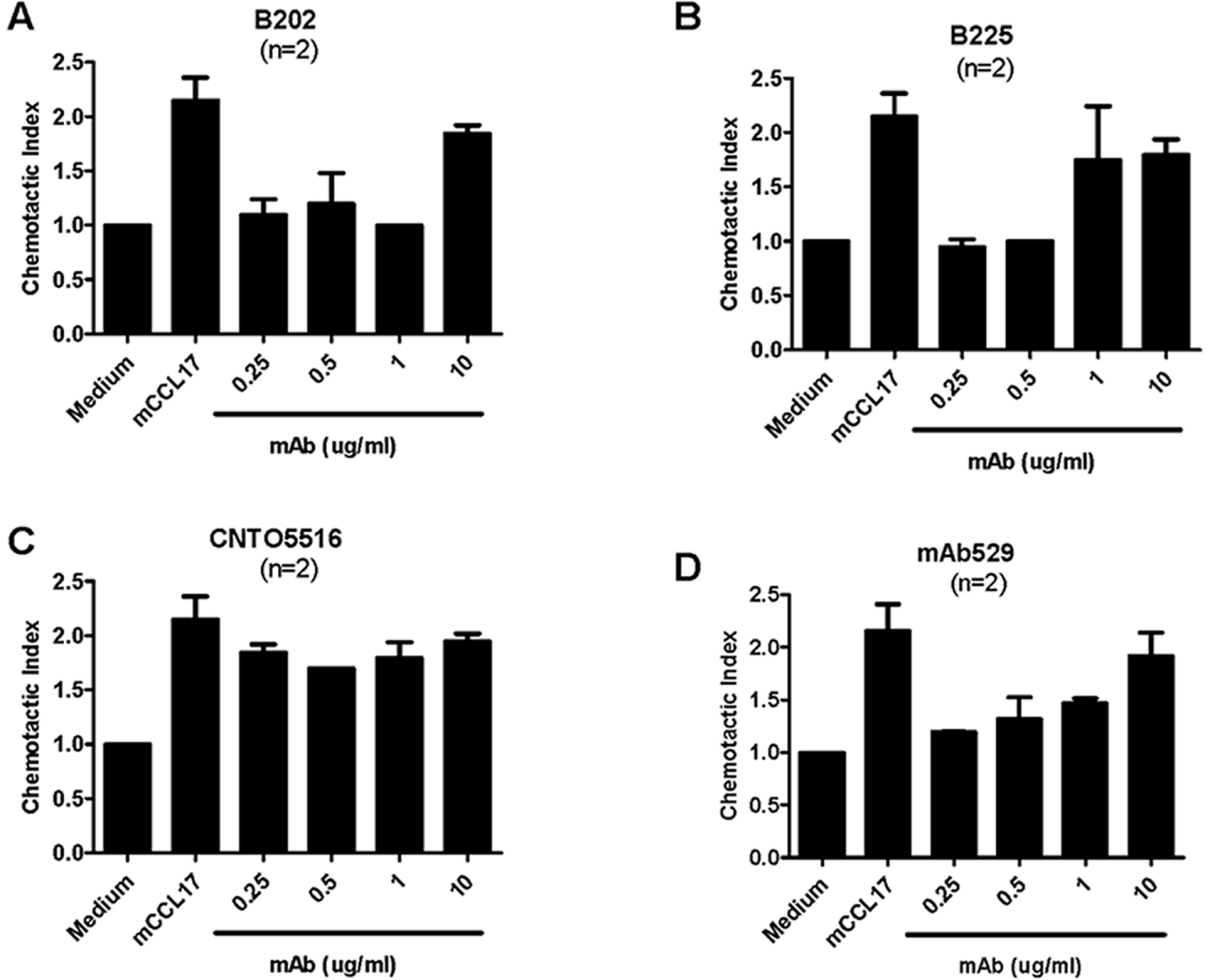
CCL17-mediated chemotaxis is inhibited in a dose-dependent manner by B202 and B225. EL4 cells were incubated with mouse CCL17 at 2/mL in the presence and absence of (A) B202, (B) B225 (C) CNTO 5516 which is an irrelevant rat:mouse chimeric antibody having a mouse IgG1 Fc or (D) MAB529 (rat antibody) which is a commercial antibody that neutralizes CCL17. Assay was repeated twice with five replicates per sample each time. Data are expressed as chemotactic index (CI = Average RLU from stimulated wells/average RLU from medium control; medium control = CI of 1). Antibody dose used for each sample is indicated along the x axis. Composite CI results from both experiments are shown.

Two neutralizing antibodies specific for CCL17 were identified from a panel and chosen for further characterization based on binding characteristics and antagonistic activity [30]. Antibody B225 was of interest because it specifically binds CCL17 whereas B202 also exhibits detectable but weak binding to CCL22. Generated in rats, both antibodies were converted to rat-mouse chimeric antibodies having rat variable regions and mouse Fc regions to facilitate *in vivo* studies. As shown in Figure 1, both antibodies effected calcium mobilization in a dose-dependent manner with the calculated IC_50_ values ranging from ∼63 ng/mL to >200 ng/mL indicating that B202 and B225 differ in effectiveness as far as blocking CCL17-mediated function. In parallel, these antibodies were assessed for the ability to block CCL17-mediated chemotaxis of the mouse EL4 T cell line. In agreement with results of calcium mobilization assays both antibodies also fully inhibited chemotaxis of EL4 cells (back to levels observed in medium alone), particularly when added at concentrations ranging from 0.25−0.5 µg/mL) (Figure 2). However, the higher concentration of 10 µg/mL of either mAb failed to block CCL17-induced EL4 chemotaxis presumably due to non-specific, IgG-mediated activation of these cells thereby promoting chemokinesis. In contrast to calcium mobilization, obvious differences between B202 and B225 in their ability to block chemotaxis could not be clearly demonstrated.

**Figure 3:**
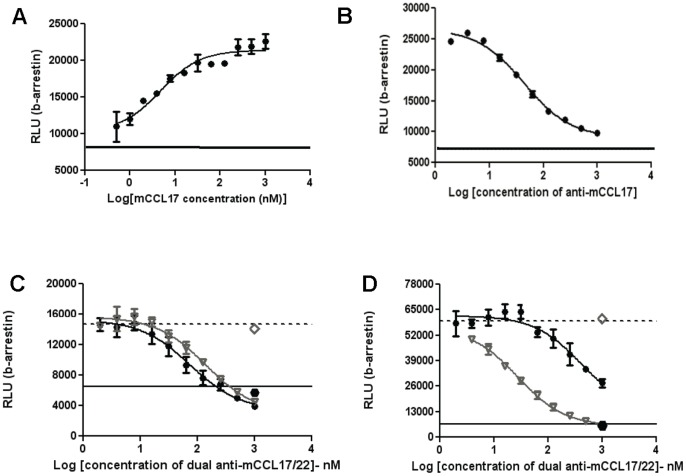
Antibodies B202 and B225 inhibit β-arrestin recruitment mediated by CCL17 interaction with CCR4. The human CCR4 β-arrestin reporter cell line was incubated with mouse CCL17 then assayed for the ability to mobilize β-arrestin as described in materials and methods. Mouse CCL17 is able to trigger β-arrestin recruitment by signaling through the human CCR4 receptor in a dose-dependent manner (A). The anti-mouse CCL17 specific antibody, B225, can inhibit mouse CCL17-induced β-arrestin recruitment (B). The dual reactive antibody, B202, strongly inhibited mCCL17 induced β-arrestin recruitment (C) while partially inhibiting mCCL22 induced β-arrestin recruitment (D). In panels C and D: B202 (*circles*); positive controls mAbs ( *inverted triangles*); isotype control (*open diamonds*); no antibody (*dotted line*); media only control (*solid line*). Data are expressed as Relative Light Units (RLU) to quantitate β-arrestin recruitment which is directly proportional to luminescent activity. Antibody dose curve starts at 1 µM with a 1∶2 dilution series (mCCL22 at 20 nM; mCCL17 at 30 nM). Antibodies were tested 3 times using CCL17 and twice using CCL22; shown here are representative data from one experiment.

To verify that these antibodies are specifically interfering with CCL17 signaling through CCR4 a reporter cell line was used to monitor β-arrestin recruitment. CCL17 functions by engaging the Gi coupled GPCR CCR4 and one of the consequences of this signaling is β-arrestin recruitment to the GPCR [16], [33], [34]. Our first challenge was to identify a reporter cell line suitable for evaluating murine CCL17 activity. Since the human cell line CCRF-CEM is responsive to murine CCL17 in the calcium flux assay, we first confirmed that mouse CCL17 could induce β-arrestin recruitment mediated by human CCR4 in a human reporter cell line (Figure 3A). Upon confirmation that murine CCL17 induced recruitment of β-arrestin in the reporter cell line, dose-response curves were performed for each of the antibodies in an effort to differentiate them according to potency (see Figure 3B for B225 and C for B202). However, both antibodies appear to perform comparably to each other in that the estimated IC_50_’s are around 100 nM. Clone B202 represents the only antibody that binds both CCL17 and CCL22 in our panel [30] so this antibody was also tested for ability to neutralize CCL22 mediated β-arrestin recruitment (Figure 3D). As shown in Figure 3, B202 is more effective at blocking CCL17 function (Figure 3C) in that only partial inhibition of β-arrestin recruitment in response to CCL22 (Figure 3D) could be demonstrated in this system.

### Both Antibodies Neutralize CCL17 Function in vivo

**Figure 4:**
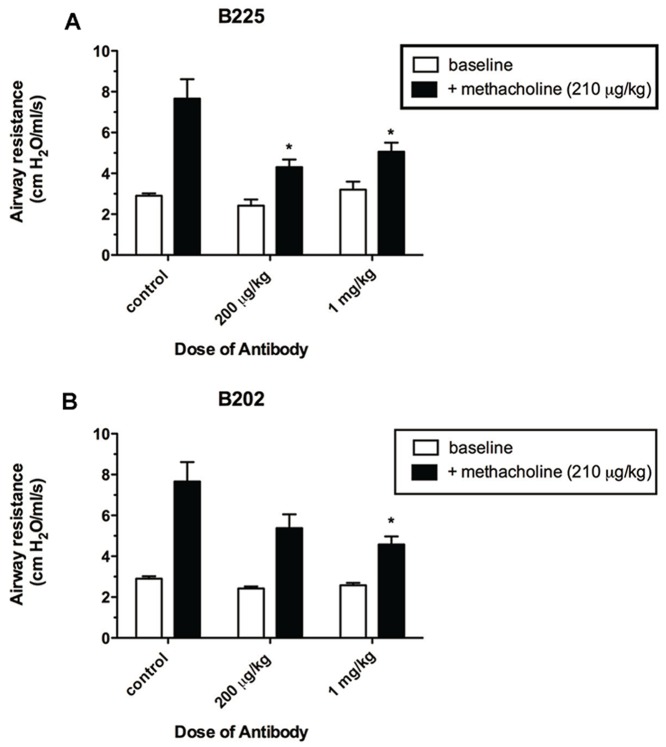
Neutralization of CCL17 in vivo ameliorates methacholine-induced AHR. Airway resistance was determined in anesthetized and ventilated mice using a Buxco plethysmograph at day 7 after conidia. Mice received IgG control or anti-CCL17 mAb at 200 µg/kg or 1 mg/kg as described in the Materials and Methods. Baseline and methacholine-induced airway resistance are shown. Data are mean ± SEM, n = 5 mice per group. *P<0.05 compared with methacholine-challenged control group (ANOVA and Student-Newman-Keuls Multiple Comparison post Test).

An *in vivo* study was run in the *A. fumigatus* chronic fungal asthma model to determine whether any difference in neutralizing activity could be seen based on targeting either CCL17 alone or targeting both CCL17 and CCL22. A role for CCL17 in fungus-induced asthma has been previously established using either CCR4 KO mice or treatment with polyclonal CCL17-specific antibodies [35]. As it has previously been shown that CCL17 levels peak at about 2 days post challenge with conidia in this model, treatment with each of the surrogate anti-CCL17 antibodies was initiated at the time of intra-tracheal challenge with live *A. fumigatus* conidia then administered every other day for 7 days as outlined in materials and methods. Following treatment, AHR in response to methacholine was assessed in all animals and as shown in Figure 4, treatment of animals with B225 resulted in statistically significant reduction in methacholine-induced AHR in animals treated with both doses tested compared to the isotype control treated group. Although treatment with B202 treatment also resulted in a reduction of AHR, the difference was statistically significant relative to the control group only at the 1 mg/kg dose.

**Figure 5:**
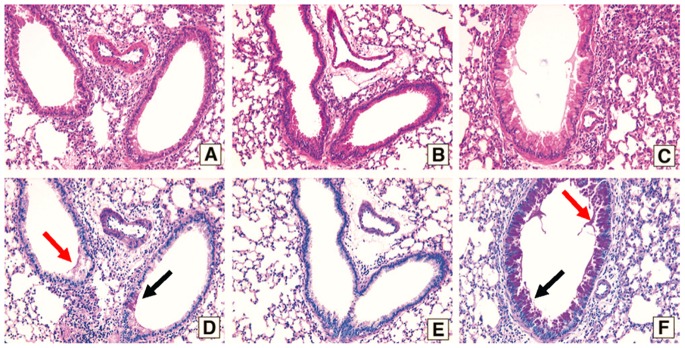
Treatment with B225 anti-CCL17 antibody inhibits inflammation and goblet cell metaplasia in the lung of A. fumigatus-sensitized and challenged mice. Histology of whole lung sections from day 7 post-conidia challenged mice treated with antibodies at 200 µg/kg. Panels A-C are H/E-stained and panels D-F are PAS-stained. In panels D and F, *black arrows* highlight goblet cells and *red arrows* point to the mucus released from these cells in the airways. Samples are from groups as follows: A and D are from isotype control treated; B and E are from B225 treated; C and F are from B202 treated mice. Representative photomicrographs of lung sections from n = 5 mice per group are shown. Original magnification was 200×.

Examination of lung tissue sections from the B225-treated mice reveals marked improvement in histology as indicated by decreased peribronchial and perivascular inflammation (H/E-stained; Figure 5B) and a lack of mucus cell metaplasia (PAS-stained cells are typically stained magenta in the airways; arrows Figure 5E) as compared to the isotype control Ig-treated group (Figure 5; panels A (H/E) & D (PAS-stained). In contrast, histological analysis of lungs from B202- treated mice showed no decrease in inflammation (compare panels A & C in Figure 5) or goblet cell metaplasia (compare panels D & E in Figure 5), despite the fact that this antibody reduced AHR comparably to treatment with B225 (see Figure 4). Rather, the B202-treated group appears to have greater PAS staining in the airway epithelial layer than either the B225- or control Ig- treated mice; a result that might reflect a tissue preparation artifact given that all animals were subjected to AHR and BALF prior to tissue fixation. In fact, PAS-stained mucus material was readily apparent in the lumen of airways in the isotype control group as shown in Figure 5, panel C.

**Figure 6:**
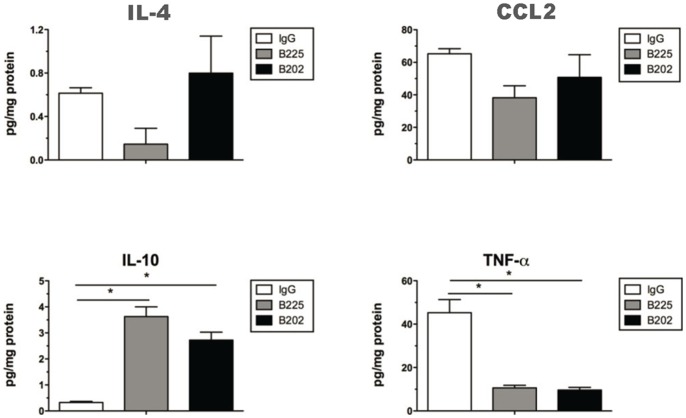
In vivo neutralization of CCL17 alters whole lung cytokine levels in A. fumigatus-sensitized and challenged mice. Cytokine and chemokine levels in homogenized whole lung samples from asthmatic mice at day 7 after conidia challenge. Bioplex analysis was used to measure cytokine and chemokine levels in these samples. Data are expressed as pg/mg for isotype control treated (*white bars*), B225 treated (*gray bars*) and B202 treated (*black bars*) Data are mean ± SEM, n = 5 mice per group. *P<0.05 compared with methacholine-challenged isotype control treated group as indicated (ANOVA and Student-Newman-Keuls Multiple Comparison post Test).

To better understand how these two antibodies are functioning *in vivo*, whole lung levels of cytokines and chemokines were profiled. Using a multiplex approach to assess cytokine and chemokine profile in whole lung, we found no difference between the three groups for a majority of factors evaluated (data not shown). However, TNF-α is significantly reduced in mice treated with either of the antibodies whereas IL-10 levels are uniformly increased compared to isotype control treated mice (Figure 6). Though not statistically significant, treatment of B225 resulted in IL-4 levels at approximately 33% of that seen in either the B202 or isotype control treated groups indicating that B202 treatment had a minor effect on IL-4 levels in the lung. Lung CCL2 levels remained relatively constant in animals treated with B202 with a slight reduction in the B225-treated group. Neither antibody impacted IL-13 or IL-5 levels in this study (data not shown). However, B225 (anti-CCL17 specific) appears to impact the response in the animal model differently than B202 as is evident in the effects on peribronchial inflammation and IL-4 production. Together, these data strongly support the hypothesis that the chimeric surrogate mAbs are blocking CCL17 function in this model.

### Variation in Neutralization Performance between the Surrogate Antibodies can be Attributed to Differences in Binding Characteristics

**Figure 7:**
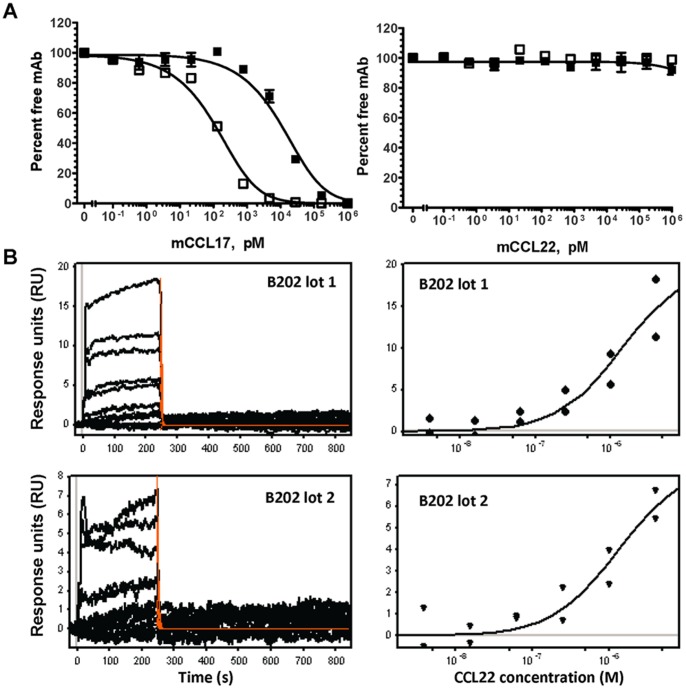
Characterization of binding properties of surrogate antibodies for CCL17 and CCL22. (A) CCL17 (*left*) but not CCL22 (*right*) can compete for CCL17 binding to B202 (*filled squares*) and B225 (*open squares*). (B) Typical Biacore sensorgrams (*left panels*) and steady-state data (*right panels*) for the interaction of two lots of B202 with murine CCL22. The interaction was analyzed in PBS pH 7.4, supplemented with 3 mM EDTA, 0.005% Tween 20 and 400 µg/mL BSA, at 25°C. In the plots, each curve (sensorgrams) and each point (steady-state plots) corresponds to a different concentration (in duplicate) of muCCL22 ranging from 3.9 nM to 4000 nM. The dissociation rates (k_d_) and affinity (K_D_) for are 0.44 and 1.4 µM; for the first lot of CCL22 (*shown in upper panels*) and the k_d_ is 0.35 and the K_D_ is 1.3 µM for the second lot of CCL22 (*shown in lower panels*).

Though mice treated with the anti-CCL17 surrogate antibodies in the *A. fumigatus* model showed improvement in asthma related symptoms *in vivo*, effectiveness of the antibodies was not equivalent *in vitro*. Since B202 binds both CCL17 and CCL22 and both ligands are produced *in vivo*, one explanation for the difference in the mouse study could be that the CCL17 binding of B202 is being divided between the two ligands. However, the results seen in the *in vitro* studies can be attributed only to CCL17. We reasoned that one factor behind this disparity could be differences in binding affinity of each of the antibodies for CCL17. To address this possibility the affinity of each mAb for CCL17 was determined using a solution equilibrium affinity assay (SEA). Binding affinity for CCL17 was 4.974±2.2 nM for B202 and 685±29 pM for B225 with the difference in affinity between the two mAbs at approximately 7-fold. Although B202 was originally identified as a potential CCL17/CCL22 dual binding antibody by ELISA (data not shown), CCL22 binding could not be clearly demonstrated using SEA in the concentration range tested (Figure 7A). Further, data shown in Figure 7 confirms that CCL22 could not compete for CCL17 binding to this antibody whereas self-competition with CCL17 could be demonstrated in a concentration-dependent manner. To clarify this apparent discrepancy in binding data for CCL22, Biacore analysis was performed to allow calculation of the relative on and off-rates for B202-CCL22 interaction. Binding of B202 to CCL22 could only be detected when the CCL22 concentration range was increased to >1 µM and this binding occurs with a very fast off-rate (Figure 7B) which would confound analysis using SEA. While B202 showed significant binding in ELISA (hybridoma screening) probably due to high mAb density on the plate which could lead to avidity or re-binding, it did not show significant binding in SEA at the concentrations tested. The avidity component is eliminated in SEA and only the intrinsic affinity plays a role in the results. In agreement with the Biacore data, detectable binding can be seen using SEA only when CCL22 is present at concentrations >100 nM (Figure 7A). This difference in binding between the antibodies for CCL17 and/or CCL22 could be a contributing factor to the difference in neutralization activity *in vivo*, particularly if the antibodies recognize the same or overlapping epitope.

**Figure 8:**
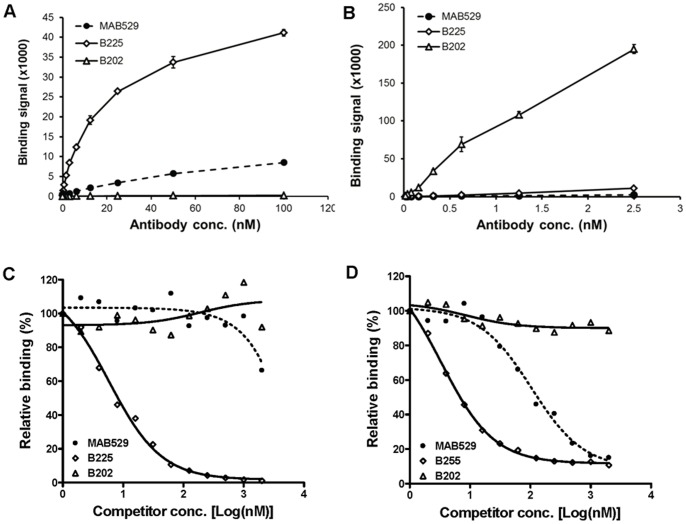
Each antibody binds a unique and non-overlapping epitope on CCL17. (A) Both B225 and MAB529 bind to mouse CCL17 proteins directly coated on microwells but B202 does not. (B) B202 binds to mouse CCL17 that has been captured by B225 indicating that B202 and B225 recognize different epitopes. (C) Labeled B225 is not competed by either B202 or MAB529 for binding to plate-bound CCL17. (D) Labeled MAB529 is competed by B225, but not B202 antibody for binding to plate-bound CCL17.

Given the size difference between the antibodies and CCL17, the prediction would be that the likelihood of two different neutralizing antibodies binding overlapping epitopes is very high. These antibodies were initially identified using a CCL17 capture ELISA to minimize epitope perturbation and favor detection of antibodies that recognize CCL17 in the most native form possible. Binding CCL17 directly to the ELISA plate could result in loss of recognizable epitopes either sequestered by binding to the plate or destroyed by denaturation. The fact that B202 and B225 have differences in CCL22 binding strongly suggests these two antibodies recognize distinct epitopes on CCL17. A series of binding experiments was performed to address this possibility. As shown in Figure 8A, the epitope recognized by mAb B225 remains available for binding when CCL17 is plate-bound; however, B202 mAb binding is completely ablated when CCL17 is presented in this way. Additional evidence that the two surrogate mAbs are recognizing different regions on CCL17 is shown in Figure 8B which shows that B202 can bind to CCL17 that is simultaneously bound to B225 using a capture ELISA. To further characterize the binding of the surrogate antibodies competition binding experiments were performed using plate-bound CCL17. In these experiments, B225 was able to efficiently compete with the control antibody, MAB529, for CCL17 binding with a predicted IC_50_ comparable to that obtained for self-competition (Figure 8C and D). As expected based on the direct ELISA binding data, B202 could not compete so no binding is seen in these competition experiments. Taken together these data indicate that B202 and B225 are binding to non-overlapping epitopes on CCL17.

## Discussion

We have generated chimeric monoclonal antibodies that neutralize mouse CCL17 through two distinct epitopes. These surrogate mAbs are inhibitory *in vitro* and *in vivo* so they can be utilized to elucidate the relative contributions of CCL17 and CCL22 in mouse models of disease. Much information regarding the receptor, CCR4, in disease conditions has been facilitated by availability of CCR4 knockout mice but these mice cannot discriminate between impact of CCL17 and CCL22 [35]–[38]. The CCL17 knock-out mice provide an opportunity to elucidate mechanism at the cellular level; however, these mice are not optimal for determining the molecular requirements for CCL17-CCR4 interaction [39]. Teasing out any differential effects associated with binding of the individual ligands to CCR4 requires CCL17 and CCL22-specific antibodies that lack cross reactivity to both. The goal of this work was to generate tools that would inhibit CCL17 function mediated by binding the CCR4 receptor thus enabling studies to understand CCL17-specific effects on CCR4 without impacting CCL22.

Treatment with neutralizing polyclonal anti-CCL17 antibodies has been reported to enhance the survival of animals with invasive *A*. *fumigatus* suggesting a prominent role for CCL17 in subverting protective immune responses in the lung [12]. By nature, polyclonal antibody preparations are comprised of many different antibodies that bind a particular antigen and it is highly likely that a subset of those antibodies cross react with other antigens that have not been identified. To minimize this uncertainty monoclonal antibodies are the preferred tool for *in vitro* and *in vivo* functional studies whenever possible. The available monoclonal antibody specific for mouse CCL17 is a rat IgG2b which behaves like mouse IgG2a *in vivo* in that it interacts with FcR to mediate effector function and antibody-dependent antigen clearance [40], [41]. The surrogate antibodies described herein have been engineered for expression as chimeric antibodies with rat variable regions and mouse IgG1 constant region. Murine IgG1 lacks effector functions as it binds FcRs with low affinity and is the least efficient at mediating effector functions of all the mouse Fc isotypes [41], [42] As a consequence, any effects seen *in vivo* in animals treated with these surrogates are most likely to be due to neutralization rather than Fc-mediated effects. It should be noted that one study uses a hamster anti-mouse monoclonal antibody but this is so far unavailable for general use [8].

Though the two surrogates, B225 and B202, as well as a commercial antibody, MAB529, all neutralize CCL17 function *in vitro*, the binding characteristics of each suggest they may be acting by different mechanisms. Only the B225 surrogate competes with the commercial mAb for CCL17 binding suggesting these two antibodies bind overlapping epitopes (Figure 8). Both antibodies also recognize CCL17 bound directly to the ELISA plate although the signal for B225 binding is on average greater than 5-fold stronger than that for the commercial antibody. Given that the binding affinity for B225 is weaker than the commercial antibody by more than 10-fold, the difference in signal is unlikely to be attributed to affinity alone. Taken together these data strongly suggest that B225 and MAB529 recognize distinct but overlapping epitopes. The second surrogate B202 appears to recognize a third epitope on CCL17 discrete from either B225 or MAB529 in that it gives no binding signal to plate-bound CCL17. Affinity for CCL17 binding by B202 is significantly weaker than either of the other antibodies yet it does not compete with B225 for CCL17 binding. This is evident in Figure 8 where it is shown that B202 binds to CCL17 presented by B225 in a capture format ELISA. In this format, no binding of B202 would be detected if the epitope for this antibody is shared by B225. However, B202 binding is readily detectable suggesting that the binding site is not obstructed by B225 binding as would be the case if the epitopes are not overlapping. Interestingly, neither MAB529 nor B202 could compete with labeled B225 for plate-bound CCL17. In fact, MAB529 binding is almost indistinguishable from B202 binding in the experiment shown in Figure 8. Using labeled MAB529 it is shown that MAB529-MAB529 self-competition was less efficient than MAB529 competition with B225 for binding to plate-bound CCL17. One possibility for these results is that the epitope recognized by MAB529 is less readily available for binding in these conditions whereas the epitope recognized by B225 remains entirely intact. In this situation, the concentration of the respective epitopes recognizable by each of the antibodies is highly biased toward B225 binding so that any competition between MAB529 and B225 binding would be masked. However, further structural and mutagenesis studies would be necessary to definitively map the epitopes.

The CCL17/CCL22 dual reactive antibody, B202, may provide some hint as to how CCL17 is interacting with CCR4. Though both antibodies inhibit CCL17 functionality, only B202 also partially inhibits CCL22 function *in vitro* (Figure 3). Both ligands function through CCR4, but share only ∼32% identity at the amino acid level; however, they have been shown to compete for CCR4 binding [14], [16], [22]. Perhaps this indicates that the CCR4 binding site involves at least 2 binding domains, and binding both sites is required to effectively engage the CCR4 signaling pathway. In this case our data could be explained if the two binding domains harbor unique epitopes that are spatially separated in the 3-dimensional structure so that mAb binding to either of the epitopes would block functional interaction with CCR4. It is possible that only one of these binding domains is conserved in both ligands in agreement with previous data showing that CCL17 and CCL22 compete for CCR4 binding [14]. To confirm the antibodies inhibit CCL17 function through CCR4, we used a reporter cell line engineered specifically to monitor CCR4 signaling by generating a chemiluminescent signal upon β-arrestin recruitment to CCR4 mediated by functional interaction with either chemokine. Treatment with either B225 or B202 inhibits β-arrestin recruitment which results in lack of chemiluminescent signal, indicating that both antibodies interfere with CCL17-CCR4 interaction. Identification of epitopes for B202 and B225 on CCL17 can provide further information regarding the role of those amino acids in mediating functional interaction with CCR4. It should be noted that all competition binding as well as functional data were generated using recombinant, full length chemokines although CCL22 can be proteolytically cleaved *in vivo* rendering it inactive [43], [44]. Whether B202 neutralizes CCL22 *in vivo* may depend on whether the epitope resides in a region on CCL22 that is available on the endogenous protein regardless of whether it is full length or proteolytically cleaved.

In support of the *in vitro* studies, preliminary data generated using B202 and B225 indicates that both antibodies also exhibit neutralizing properties *in vivo* in the fungal asthma model. This is evident in mice treated with surrogate antibodies as they exhibited less airway hyperresponsiveness upon methacholine challenge compared to mice treated with isotype control. The *in vivo* data reported herein, appear to indicate that reduced AHR in the *A.fumigatus* model observed using a rat IgG2 anti-CCL17 antibody was likely due to neutralization of CCL17 rather than immune clearance of the Ab:CCL17 complex by an anti-rat Ig response [11]. At the genetic level homology between mouse IgG2 and Rat IgG2b Fc is very high so that the rat anti-CCL17 mAb could potentially mediate clearance through the FcR; however, the Fc portion of the rat antibody is unlikely to be immunogenic [45]. The commercial mAb MAB529 that neutralizes CCL17 chemotactic activity *in vitro* is a rat IgG2b which, in the mouse, behaves comparably to mouse IgG2a in mediating effects through FcR binding and potentially confounding data interpretation [41]. The chimeric mAbs described herein are expressed as mouse IgG1 and possess minimal effector function since this isotype preferentially binds to low affinity FcRs, so the likelihood that there are Fc-mediated affects is also minimal [41], [42]. This may be especially important in that a role for Fc signaling in regulating CCL17 levels has been suggested in RA patients [46]. We have chosen to generate chimeric antibodies, that are as murine as possible, even though the duration of treatment in this experiment is brief making it unlikely the mice are mounting a significant anti-rat protein response. By using the chimeric B202 and B225 mAbs we believe that the *in vivo* effects can unequivocally be attributed to neutralization of CCL17.

That B202 and B225 exhibited different effects *in vivo* may have been predicted by the performance *in vitro*. Figure 1 illustrates that B225 inhibition of CCL17-mediated calcium mobilization in CCRF-CEM cells appears more effective than B202 by ∼3.5-fold based on the calculated IC_50_’s. Perhaps this can be accounted for by the difference in binding affinity between the antibodies which is calculated to be on the order of ∼7-fold. In addition to affinity, these antibodies differ in recognition of CCL17 in that they are binding to non-overlapping epitopes and this feature should also be taken into consideration especially given that only B202 can be demonstrated to bind CCL22. Taking into account the differences in recognition of CCL22, binding affinity and epitope recognition it is likely that all attributes feature in the results obtained *in vivo*. An important question that is not addressed in the mouse study presented herein is whether these two antibodies have different effects on the cell populations involved in the inflammatory response. Of particular interest is Treg function since it has been reported that CCL17 and CCL22 may differentially affect this population [9], [24], [25]. Though the work shown here hints at mechanistic differences, additional animal studies will be necessary to elucidate the details as to how B202 and B225 function *in vivo*.

We cannot rule out the possibility that *in vivo* our antibodies impact CCL17 signaling through a receptor other than CCR4 either directly or indirectly. CCR8 has been reported as an additional receptor for CCL17 and CCL22 but this finding could not be confirmed [47], [48]. A recent report demonstrated reduced migration of CCL17 KO cells in response to CCL19, CCL21 and CXCL12 corresponding to ligands for CCR7 and CXCR4, and this effect was independent of CCR4 expression [39]. One explanation for this result could be that CCL17 interacts with CCR7 and CXCR4 to mediate function in a context different than their known ligands. Alternatively, it is possible that CCL17 could impact signaling through CCR7, CXCR4 or even other receptors via indirect mechanisms, for example by interaction with other chemokines, to enhance or reduce signaling. Though not reported for CCL17, CCL22 has been demonstrated to interact with a number of chemokines including CXCL10, CCL19 and CXCL12 [49]. Perhaps one or both of our antibodies are blocking a site necessary for interaction with another chemokine thereby diminishing any enhancing effect conferred upon or by CCL17. Any potential effects of the antibodies on CCL17 via a receptor other than CCR4 would not be evident here in that the CCR4 reporter cell line that was used does not express either CCR7 or CXCR4. To fully understand CCL17 function, investigation into the potential for CCL17 to interact with other chemokines may be as informative as looking for other receptors.

Using chimeric neutralizing surrogate antibodies, we have demonstrated that CCL17 harbors at least two distinct binding sites both of which are critical for mediating function. Interference with either site is sufficient to inhibit activity both *in vitro* and *in vivo*. Though both antibodies neutralize CCL17 activity, B202 also binds, and partially inhibits function of, CCL22 suggesting that at least one of the binding sites is shared between CCL17 and CCL22. Since our interest was in antibodies specific for CCL17 only, no strategy was employed to enrich the panel for antibodies that bound both CCL17 and CCL22. In fact, B202 binds CCL22 with very poor binding affinity which may explain the partial neutralizing effect on CCL22 activity. These antibodies offer the opportunity to better differentiate CCL17 and CCL22 function both at the cellular and biochemical level.
